# ECG features and methods for automatic classification of ventricular premature and ischemic heartbeats: A comprehensive experimental study

**DOI:** 10.1038/s41598-017-10942-6

**Published:** 2017-09-11

**Authors:** Lucie Maršánová, Marina Ronzhina, Radovan Smíšek, Martin Vítek, Andrea Němcová, Lukas Smital, Marie Nováková

**Affiliations:** 1 Department of Biomedical Engineering, Faculty of Electrical Engineering and Communication, Brno University of Technology, Technická 12, Brno, 616 00 Czech Republic; 2 Department of Physiology, Faculty of Medicine, Masaryk University, Kamenice 5, Brno, 625 00 Czech Republic; 3 Institute of Scientific Instruments, The Czech Academy of Sciences, Královopolská 147, Brno, 612 64 Czech Republic

## Abstract

Accurate detection of cardiac pathological events is an important part of electrocardiogram (ECG) evaluation and subsequent correct treatment of the patient. The paper introduces the results of a complex study, where various aspects of automatic classification of various heartbeat types have been addressed. Particularly, non-ischemic, ischemic (of two different grades) and subsequent ventricular premature beats were classified in this combination for the first time. ECGs recorded in rabbit isolated hearts under non-ischemic and ischemic conditions were used for analysis. Various morphological and spectral features (both commonly used and newly proposed) as well as classification models were tested on the same data set. It was found that: a) morphological features are generally more suitable than spectral ones; b) successful results (accuracy up to 98.3% and 96.2% for morphological and spectral features, respectively) can be achieved using features calculated without time-consuming delineation of QRS-T segment; c) use of reduced number of features (3 to 14 features) for model training allows achieving similar or even better performance as compared to the whole feature sets (10 to 29 features); d) k-nearest neighbours and support vector machine seem to be the most appropriate models (accuracy up to 98.6% and 93.5%, respectively).

## Introduction

Cardiovascular diseases are currently the most common cause of death worldwide^1^. Due to simplicity of the method, its non-invasive character and cheapness of the procedure, electrocardiography is still the most available and widely used method for the heart electrical activity examination. Electrocardiogram (ECG) reflects the electrical activity of the heart and gives a lot of information about heart function required for diagnosis of various diseases. Automatic analysis of ECG is a fundamental task in cardiac monitoring, especially in case of long-term monitoring, where large amount of data is recorded^1, 2^. Manual evaluation of such data is extremely time-consuming. Therefore, there is an effort to improve conventional and develop new methods for processing and analysing of electrocardiographic records.

Automatic classification of the heartbeats is one of the most important steps towards the identification of pathology using ECG. The correct choice of classification algorithm and features representing heartbeats is crucial for successful classification. Although many methods have been reported, their direct comparison is questionable due to their differences in: a) types of heartbeats being classified (normal *vs* ischemic^3–6^, normal *vs* ventricular premature beats – VPBs^7–14^ etc.); b) ECG features (morphological^4, 5, 7, 15, 16^ spectral^8, 14^ first-order or higher order statistics^10, 12^ non-linear^9^, heart-vector projection^17^ etc.); c) classification models (discriminant function^4, 9^ cluster analysis^14^, artificial neural network^18^, naive Bayes classifier^13^, support vector machine^6, 7^ k-nearest neighbors^5, 11^ etc.).

In this study, various approaches were applied for classification of heartbeats derived from experimental data recorded in rabbit isolated hearts under myocardial ischemia. In such experiments, the progression of myocardial ischemia and subsequent arrhythmias (ventricular premature beats – VPB, supraventricular premature beats, atrioventricular blockades, etc.) can be successfully evaluated. Four types of heartbeats were recognized: non-ischemic sinus, moderate ischemic, severe ischemic, and VPB. Moderate and severe ischemia as well as VPBs dramatically affect QRS complex morphology. On the other hand, QRS morphology in all these situations can be identical. It may result in reduced performance of classification due to decreased ability of the features to discriminate between these heartbeat types. Such phenomenon has not been covered by previous studies and, therefore, it is desirable to focus on it.

In this study, the most frequently used classification models (discriminant function analysis, naive Bayes classifier, support vector machine, and k-nearest neighbours – all with several different settings) and features (morphological and spectral) were tested on the same data set. Such approach allows reliable comparison of their suitability for classification. Besides commonly used voltage-related and interval-related features (such as ST segment deviation, QRS complex duration, etc.), new features based on area under various parts of ECG and its spectral representations obtained by four different approaches (fast Fourier transform, short-time Fourier transform, continuous wavelet transform, and Wigner-Ville distribution) were proposed. Finally, the effect of ECG segments definition (used for feature calculation) on classification performance was evaluated.

## Methods

**Figure 1:**
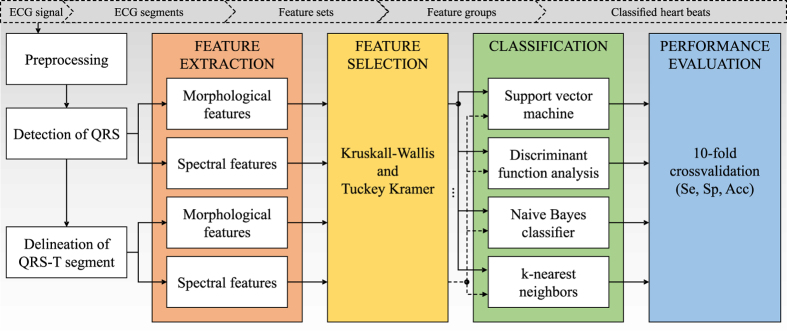
Complete scheme of electrogram processing and heartbeat classification.

This section introduces proposed methods, including data acquisition, signal processing, feature extraction, feature number reduction, and classification (see Fig. 1).

### Experimental data

The pathologies, that are classified in this work are not present in ECG signals from publicly available standard databases^19–21^ therefore it was necessary to provide experimental ECG recordings. All experiments were carried out with respect to recommendations of the European Community Guide for the Care and Use of Laboratory Animals and according to the experimental protocol approved by the Committee for Ensuring the Welfare of Experimental Animals, Faculty of Medicine, Masaryk University.

**Figure 2:**
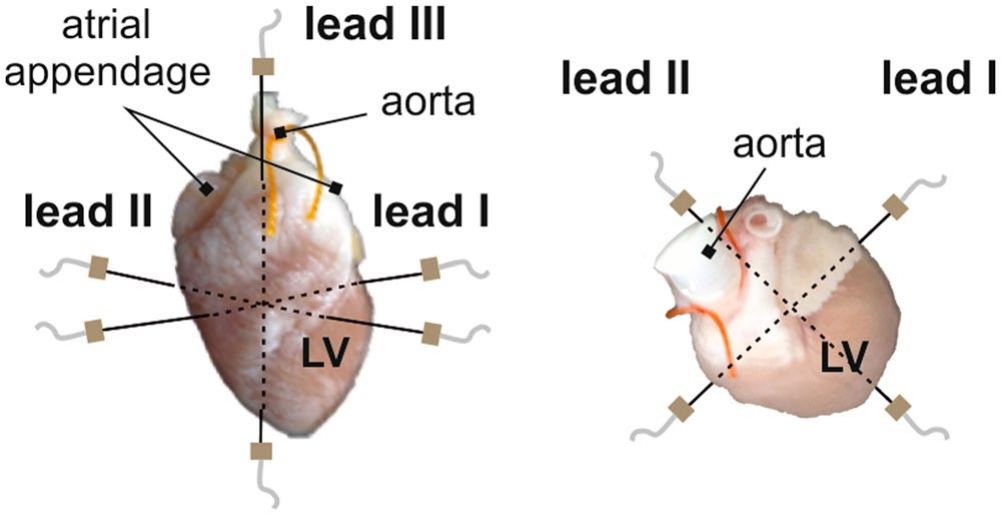
Front (left) and top (right) view of orthogonal system of electrodes. LV – left ventricle.

Data were recorded during experiments focused on the effects of global ischemia on cardiac activity. The isolated hearts of 21 New Zealand rabbits perfused with Krebs-Henseleit solution (1.25 mM Ca^2+^, 37 °C) according to Langendorff with constant perfusion pressure (80 mmHg) were used in the study. During all experiments, ECGs were recorded by touch-less method using the orthogonal lead system which includes three pairs of Ag-AgCl disc electrodes (see Fig. 2). After stabilization period (30 min), global ischemia (10 min) followed by reperfusion (10 min) were carried out. More detailed information about experimental setup and data recording according to this protocol can be found in ref. 22. The sampling frequency of 2 kHz and 16 bit resolution were used, which are sufficient for further correct detection of QRS complex and delineation of QRS-T segment.

### ECG processing

The low-frequency baseline wandering was suppressed by zero-phase Lynn’s filter with cut-off frequency 0.5 Hz. Then, QRS complexes were automatically detected by algorithm based on wavelet transformation using biorthogonal wavelet bior1.5. According to the detected QRS positions, 280 ms long QRS-T segments (part of a signal 30 ms before and 250 ms after QRS positions) were selected. Then, manual delineation of QRS-T was performed regarding three-lead ECG, including detection of the beginning of QRS, J point (QRS offset) and the end of T wave. After that, manual classification of heartbeats type (selected QRS-T) was performed by an expert. ECG from lead III was excluded from further analysis due to frequent occurrence of movement artefacts.

### Heartbeat types

The narrow QRS complexes are related to electrical impulse generated by the sinus node and physiologically conducted through the ventricles. Myocardial ischemia and concomitant disorders lead to the reduced impulse propagation velocity and the changes in path of propagation due to presence of ectopic centres or blocked regions in the ventricles. These changes are reflected in ECG mainly as QRS widening, ST segment deviation (depression or elevation) and T wave polarity inversion, such as in case of ischemia manifestation in human ECG^23^. Onset of the changes corresponds with the 3^rd^–5^th^ minute of ischemic period and their magnitude depends on severity of myocardial ischemia. Therefore, four types of heartbeats with different morphology were classified in this study:

- non-ischemic (NOR) selected from stabilization period,
- VPBs selected from ischemic period,
- moderate ischemic beats (ISM) selected from ECG recorded in the 5^th^ minute of ischemia, where only slight changes are characteristic for ECG morphology,
- severe ischemic beats (ISE) selected from ECG recorded in the 10^th^ minute of ischemia, where the most prominent changes in ECG morphology are present.

**Figure 3:**
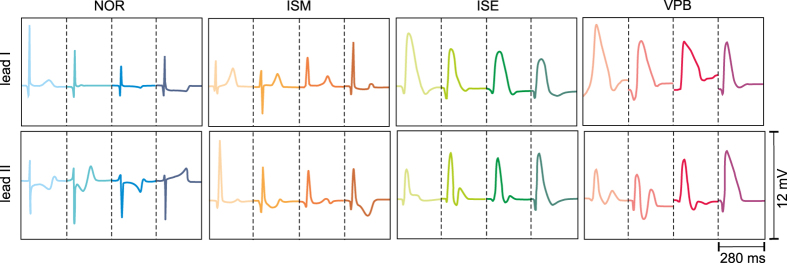
Types of classified QRS-T segments. Segments from four different experiments are shown in each group. NOR, ISM, ISE, VPB – non-ischemic, moderate and severe ischemic beats and ventricular premature beats, respectively.

Totally more than 260 000 heartbeats were labelled. However, only 172 VPBs were found in the records. Therefore, 220 representatives from each group (NOR, ISM and ISE) were selected to reduce the imbalance between classes and to provide sufficiently large data set for successful classification at the same time. In Fig. 3, all types of QRS-T segments selected from four different experiments are shown. It is evident, that NOR and ISM from lead I as well as ISE and VPB from leads I and II are quite similar. On the contrary, there are significant differences between beats from the middle (ISM) and the end (ISE) of ischemic period on one side and ISM and VPB on the other side. Besides mentioned inter-class differences, the intra-class variability (mainly regarding R peak deviation) is quite high.

### Features calculation

Commonly used (e.g refs 6, 10, 14 and 15) and some newly proposed morphological and spectral features were calculated from each selected QRS-T segment.

### Morphological features: calculation from QRS-T obtained from ECG delineation

Total 71 features representing the ECG morphology were calculated from the segments selected from lead I and lead II based on the results of previous delineation (QRS onset, QRS offset (J point) and T wave offset).

First type of features represents *interval* (QRS, QT and ST-T duration) and *voltage* (e.g. maximal absolute, positive and negative deviation of QRS, maximal deviation of ST-T interval, deviation of ST segment 20 ms after QRS offset) characteristics of QRS-T. Illustration of selected features from this group is in Fig. 1a.

**Figure 4:**
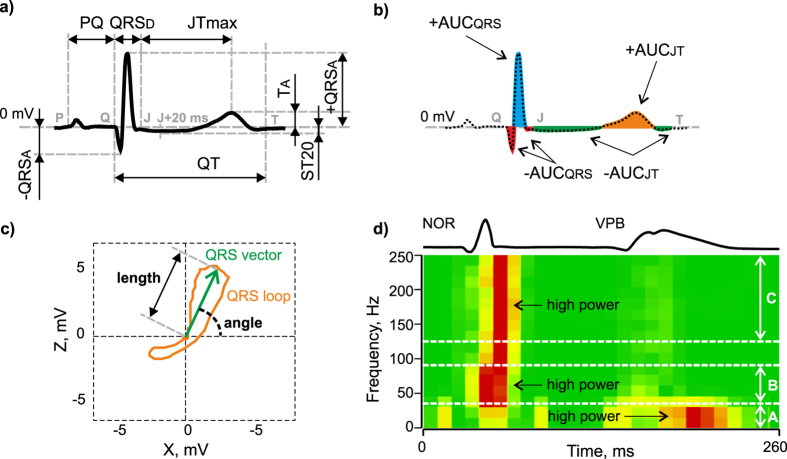
Selected features definition: (**a**) interval (QRS_D_, QT, PQ, and JTmax represent the duration of QRS complex, QT interval, PQ interval, and segment between J point and maximal deviation of T wave, respectively) and voltage (+QRS_A_, −QRS_A_, T_A_, and ST20 represent maximal positive deviation of QRS, maximal negative deviation of QRS, maximal deviation of ST-T interval, and deviation of ST segment 20 ms after QRS offset, respectively) characteristics of ECG; (**b**) areas under various parts of QRS-T (−AUC_QRS_, +AUC_QRS_, −AUC_JT_, and +AUC_JT_ represent area under negative and positive part of QRS and negative and positive part of ST-T interval, respectively); (**c**) 2D QRS loop parameters (length and angle of maximal electrical vector of QRS in horizontal plane); (**d**) spectrogram of QRS used for calculation of (sum of frequency power (normalized for each frequency component separately - created by^28^) in three bands (A – 0–35 Hz, B – 35–90 Hz and C – 125–250 Hz); NOR, VPB - normal and ventricular premature beats, respectively.

Second type of features describes *areas under* (AUC) various *parts* of QRS-T (e.g. AUC of the whole QRS-T, QRS and of only negative part of ST-T interval). Additionally, values of all AUC based features relative to AUCqrst and positive to negative AUC ratios were calculated. Examples of this type of features are shown in Fig. 4b.

The last type of features was calculated from *2D QRS* and *ST-T loops* in horizontal plane: angle and length of maximal electrical vector separately for each loop. Examples of selected features are shown in Fig. 4c.

Interval characteristics and 2D loops features (total 7 features) were calculated using both leads together; other 32 features were derived for each lead separately.

### Morphological features: calculation from segments obtained from R peak detection

Total 44 features were calculated from the segments obtained from R peak positions. These features do not depend on manual delineation procedure. Thus, their extraction is easier, faster and more objective than in previous case.

The first type of features represents *value* and *position* of maximum and minimum deviation of the whole 280 ms long QRS-T. Other features were computed as a difference between the deviations and as a time interval between their positions.

The second type of features is based on *AUC* calculated from the segments defined as < R-*t*, R + *t* > , where R is the position of detected R peak and *t* = 40 ms, 60 ms or 100 ms. Values of *t* were chosen according to RR, QRS and QT intervals reaching in stabilization 344 ± 46 ms, 24 ± 4 ms and 175 ± 22 ms, respectively. Thus, the segments selected using different *t* contain QRS, QRS with adjacent ST or almost the whole QRS-T, respectively. Corresponding features include information about different parts of QRS-T, which may affect their discriminating ability and the performance of heartbeat classification. Under ischemia, RR and QRS are prolonged, whereas QT is shortened. Hence, selected segments do not contain the parts of adjacent heartbeats (even in case of the highest *t*), which is important for accurate features calculation.

AUC was also calculated from the whole 280 ms long QRS-T segments containing QRS-T and a short part of isoline after T wave and from negative and positive parts of the segments separately. Relative values and ratios of AUC features were calculated, too.

### Spectral features: calculation from QRS obtained from ECG delineation

Total number of 24 features representing the spectrum of QRS was calculated from both leads (12 features for each lead). QRS were selected based on the manually detected QRS onsets and J points.

The first type of features was computed from spectrum obtained by *fast Fourier transform* (FFT) as the sum of particular components in three frequency bands commonly used for analysis of conventional and high-frequency QRS^19^: 0–35 Hz, 35–90 Hz and 125–250 Hz.

The second type was defined as mean, median and maximum of spectrogram computed by *short-time Fourier transform* (STFT). Only non-zero frequency components were included in calculations.

The third type of features was extracted in time-scale domain by *continuous wavelet transform* (CWT) performed using symlet wavelet sym2 at all integer scales from 1 to 32. Besides suitability for CWT, sym2 is symmetrical and allows achieving good results in case of noisy signals^24–26^, Selected scales correspond to frequency range of the signal. The first two features were calculated as mean and maximum of CWT matrix. The third feature was found as a mean correlation between QRS and scaled versions of the mother wavelet placed in such a way that the highest correlation was obtained. On the contrary, the last feature was represented by mean correlation between QRS and differently placed wavelet scaled in such a way that the highest correlation was obtained.

The last features group was calculated from *Wigner-Ville distribution* of QRS. The features were calculated as a maximum and mean value of QRS distribution in the frequency range 0–500 Hz.

### Spectral features: calculation from segments obtained from R peak detection

Total 24 features representing ECG spectrum were calculated from each type of segments defined as < R-*t*, R + *t* > , where *t* = 20 ms, 30 ms or 50 ms. As compared to morphological features, narrower boundaries were set in order to select segments containing mainly QRS (narrow in case of non-ischemic condition or prolonged one in case of ISE or VPB). This is in agreement with well known approach, where spectral content of QRS complex (not ST-T) is used to assess myocardial ischemia in electrocardiographic signals^27^. Generally, the features were computed by similar way as in previous section. Selected features are shown in Fig. 4d.

### Features number reduction

Training of classification model using high-dimensional feature set may lead to model overfitting^29^. Therefore, so called filter method (e.g. refs 29 and 30) was applied to select only the most informative features showing statistically significant differences among particular classification groups. At first, the Shapiro-Wilk test was used to reveal data distribution. Then, non-parametric Kruskal-Wallis test (α = 0.05) followed by Tukey-Kramer post-hoc test were used to compare features from particular groups and indicate those suitable for discrimination between various heartbeat types. Only features with significant differences between all pairs of classification groups were used for further analysis.

### Automatic classification

Four models (with various settings) were used for automatic heartbeat classification based on selected features^29, 31, 32^:

- discriminant function analysis (DFA) with linear and quadratic function,
- naive Bayes (NB) classifier with Gaussian kernel and kernel density function estimation,
- support vector machine (SVM) (one-vs-all approach for multiclass approach) of general type and with radial basis function (RBF),
- k-nearest neighbors (k-NN) with different *k* value (*k* = 1, 5, 10).

Selected models are widely used for heartbeat classification (see below). Moreover, the models represent different types of classifiers^32^: a) supervised parametric (DFA and NB); b) supervised non-parametric (SVM); c) non-parametric algorithm based on instance learning (k-NN).

Training and testing of all classifiers were performed using 10-fold cross-validation approach^31^. Standardized features were used as an input to classification models. Common standardization procedure (e.g. ref. 33) of training and testing data was performed for each fold separately, based on statistical measures (mean and standard deviation) calculated from training instances.

Classification performance of each approach was evaluated by mean overall accuracy (Acc), where Acc of particular validation folds was defined as a number of correctly classified heartbeats in all groups related to their total number. Sensitivity (Se) and specificity (Sp) were also calculated for more detailed performance analysis^34^.

## Results and Discussion

### Selected classification features

**Figure 5:**
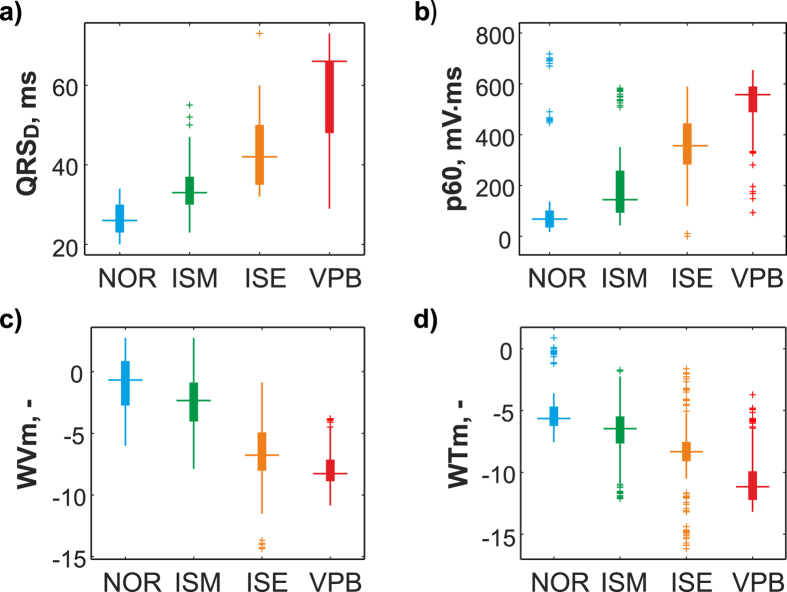
Distribution of selected features with significantly different values among all types of heartbeats confirmed by statistical analysis (p < 0.05): (**a**) QRS complex duration (QRS_D_), (**b**) area under segment selected as a part of ECG 60 ms before to 60 ms after R peak position (p60), (**c**) mean value of QRS in Wigner-Ville distribution within frequency range 0–500 Hz (WVm), (**d**) mean of continuous wavelet representation of QRS complex (WTm).

Based on the post-hoc test results, only the most informative features were chosen from the whole data set to represent QRS-T segments. Total number of 26, 24, 10, and 29 features were selected from morphological group obtained from ECG delineation and R peak detection and spectral group obtained from manual delineation and automatic R peak detection, respectively. Distribution of four selected features in particular classification groups is shown in Fig. 5.

For more detailed evaluation of suitability of particular features for heartbeat classification, main feature groups were additionally divided into subgroups according to their characteristics as follows:

- 26 morphological features obtained from delineation (MorphD) were divided into 5 sets: CommonD (interval and voltage-based characteristics, n = 3), LoopD (features from 2D loops, n = 2), AreaD (general AUC based features, n = 9), AreaDa (AUC based features calculated from absolute values of the segments, n = 5), and AreaDr (relative AUC based features, n = 7);
- 24 morphological features obtained from R peak detection (MorphR) were divided into 3 subgroups: CommonR (interval and voltage-based features, n = 3), AreaR (general AUC based features, n = 16) and AreaRa (AUC based features calculated from absolute values of the segments, n = 5);
- 10 spectral features obtained from delineation (SpectralD) were not divided because of low number of features;
- 29 spectral features obtained from R peak detection (SpectralR) were divided into 3 subgroups according to *t* used for segments definition (see above): S20 (n = 6), S30 (n = 9) and S50 (n = 14) for t = 20 ms, 30 ms and 50 ms, respectively.

### Evaluation of classification performance

Mean overall Acc for different classification approaches (i.e. different features groups, classification models and settings) are summarized in Table 1.

**Table 1:** Mean accuracies (%, for 10-fold cross-validation) of various approaches for heartbeat classification.

Feature groups and subgroups	Classification model								
	k-NN			DFA		NB		SVM	
	k = 1	k = 5	k = 10	Linear	Quadratic	Gaussian	Kernel	RBF	Linear
CommonD	98.5	97.7	91.9	75.6	77.1	77.7	81.2	81.6	79.6
LoopD	97.6	94.8	87.6	63.1	60.7	59.6	80.6	66.3	64.6
AreaD	99.0	98.6	93.6	81.7	75.9	75.8	80.7	86.9	83.9
AreaDa	98.9	96.7	90.6	72.0	72.8	72.7	78.4	75.8	75.0
AreaDr	98.3	96.1	90.3	62.6	65.8	66.1	80.1	81.9	78.1
MorphD	99.0	98.0	94.2	77.9	74.0	74.4	83.5	93.5	87.9
CommonR	98.4	97.3	93.5	77.1	62.0	62.0	79.4	88.8	82.3
AreaR	98.2	94.2	88.7	50.3	56.0	57.3	83.2	63.3	61.7
AreaRa	99.0	98.3	91.6	68.1	66.3	66.4	83.4	85.7	80.7
MorphR	71.7	77.0	76.8	47.2	57.3	57.3	70.8	46.5	46.3
SpectralD	94.8	93.2	90.0	62.6	45.1	45.4	82.8	72.7	69.4
S20	98.8	96.1	92.0	50.4	23.3	23.5	83.5	79.6	61.7
S30	97.1	93.0	89.1	58.8	37.6	37.9	84.2	74.2	70.5
S50	98.0	95.8	91.1	60.3	34.7	34.8	87.4	82.0	79.2
SpectralR	97.3	96.2	91.2	50.6	33.9	34.0	86.1	82.1	80.1

At first, use of *various features groups* for classification was evaluated. Higher Acc was generally obtained using morphological features as compared to spectral ones; the difference reached about 38% in case of Gaussian NB model (compare equally sized AreaD and S30 in Table 1). This observation is in accordance with the results reported previously, where VPBs (widely variable, bizarre waveforms) were better recognized by morphological features in contrast to NOR, blocks and paced beats (i.e. repeating waveforms), which were successfully classified by time-frequency ones^35^.

Among various features subgroups, CommonD, AreaD, CommonR, AreaRa, and S50 allow achieving quite high Acc with the most of presented classification models and, therefore, seem to be the most suitable ones. Furthermore, abovementioned area based and spectral subgroups provided successful results with three classification models in this study. Computing of area based features takes into account segment’s curvature, duration and sometimes (in case of AreaD) polarity. Consequently, minor changes in ECG morphology result in prominent changes of area based features and, thus, can be more easily detected by these features as compared to voltage or interval characteristics of the segments. It is obvious that 100 ms long segments (*t* = 50 ms) are more suitable than shorter ones. Features calculated from longer segments (S50) reflect the most prominent ischemic changes both in QRS (prolongation and elevation) and ST (depression or elevation) (see Fig. 3).

The results of our study confirm that use of large features group is not necessarily the best choice. Using particular subgroups consisting of less number of features reveals comparable or even better results when compared with classification based on the whole features group. This is applicable in all models presented in this study. Our data correspond with the results of other authors^12^, where the improvement of performance indices (Se by 9% and Sp by 1%) was achieved by reducing of feature set (28 features from 90) using wrapper technique. In another study^10^, use of the whole set of 50 features improved the results obtained by 2 features (selected by forward-backward selection) only by 10%.

Classification performance of the methods utilizing features obtained from delineation was usually higher than in case of ‘artificial’ segments obtained from R peak detection. However, the advantage of the former approach is not so significant. Furthermore, classification by NB and SVM using spectral features calculated from strictly defined segments was more successful as compared to commonly computed features (compare SpectralR and SpectralD in Table 1). Thus, sufficient classification can be performed using features derived directly from ECG without previous time-consuming delineation. It should be noted that this would be valid not only for manual but also for automatic delineation, which is challenge task itself^36^.

At second, *different classification models* were compared. The best results were generally obtained by k-NN classifier in the whole test range of k-values. Corresponding Acc was in the range of 71.7–99.0% (with more than a half of Acc values over 90%, see Table 1). Quite lower performance was obtained by kernel NB and RBF SVM (mean Acc through all features subgroups of 81.7% and 77.7%, respectively). Linear SVM allowed achieving even poorer results (mean Acc of 73.6%) which corresponds with the results of previous study where higher Acc (by approx. 4%) was provided by RBF SVM in comparison with linear one^5^. The lowest overall classification performance was obtained by Gaussian NB (mean Acc of 56.3%) and DFA (mean Acc of approx. 60% for both settings).

Furthermore, Gaussian NB and quadratic DFA seem to be sensitive to the type of classification features; they are characterized by Acc significantly varying (both in the range of approx. 23–77%) among various feature groups used for model training.

Performance indexes for classification approaches providing the best results (see Table 1) are summarized in Table 2. For each classification model, two best features subgroups were selected. The whole features groups significantly larger than particular subgroups were excluded from the comparison. The k-NN and RBF SVM trained on area based or common features are evidently the most suitable for heartbeat classification with both Se and Sp higher than 80% in all classification groups. It should be noted that k-NN with k = 5 is suggested instead of k = 1 (both provide similar performance, see Table 1) to avoid overfitting and loss of generalization ability of the model^29, 31^. Although Acc for RBF SVM and kernel NB are very similar (in the range of 83–88%), Se obtained in ISM and ISE and Sp obtained in VPB of the second model are lower than corresponding performance indices of SVM. On the contrary, despite lower Acc of DFA as compared to NB, Se and Sp obtained for particular heartbeat types by former model were more successful (especially in case of CommonR features, where all performance indices are exceeding 68%) than those of the latter (with some Se lower than 45%). Reduced performance provided by DFA and NB may be explained by high number of misclassifications (i.e. false positives or negatives) associated with the reduced ability of the features to discriminate between: a) moderate ischemic and non-ischemic beats; b) severe ischemic and moderate ischemic beats; c) severe ischemic and ventricular premature beats. In Table 3, confusion matrix for linear DFA trained on CommonR features is shown. As expected, detection of pathological heartbeats is rather difficult due to similarity in their morphology (see Fig. 3). Particularly, ISM beats were often classified as NOR, ISE were assigned as NOR or ISM and VPBs were inaccurately recognized as ISE. Non-ischemic segments differ significantly from the others and, consequently, were classified successfully (with only 16 segments from total 220 misclassified as ISM).

**Table 2:** Mean performance indices of the best classification approaches (in %, for 10-cross validation). Se, Sp, Acc – sensitivity, specificity and accuracy, respectively (in %, for 10-cross validation).

Classifier	Heartbeat	Se	Sp	Acc	Classifier	Heartbeat	Se	Sp	Acc
k-NN	NOR	100	99.8	98.6	k-NN	NOR	100	100	98.3
k = 5	ISM	97.7	99.3		k = 5	ISM	98.2	99.2	
AreaD	ISE	98.2	98.5		AreaRa	ISE	97.7	98.4	
	VPB	95.9	99.7			VPB	95.6	99.8	
SVM	NOR	92.7	92.6	88.8	SVM	NOR	93.3	92.8	86.9
RBF	ISM	80.9	95.4		RBF	ISM	82.3	95.1	
CommonR	ISE	90	96.9		AreaD	ISE	85	95.4	
	VPB	92.4	99.8			VPB	87.8	98.9	
NB	NOR	72.7	98.7	87.4	NB	NOR	97.7	82.4	83.4
Kernel	ISM	45	73		Kernel	ISM	41.3	95.1	
S50	ISE	68.2	79.4		AreaRa	ISE	38.6	97.2	
	VPB	93	63.2			VPB	94.1	82.1	
DFA	NOR	100	82.2	81.7	DFA	NOR	91.4	95.5	77.1
Linear	ISM	49.1	97.2		Linear	ISM	73.3	89.5	
AreaD	ISE	79.5	90.8		CommonR	ISE	68.6	86.8	
	VPB	75	93.7			VPB	75.6	94.5

**Table 3:** Confusion matrix for linear DFA classification using CommonR features (‘cumulated’ through cross-validation).

	Classifier output			
	NOR	ISM	ISE	VPB
Real output				
NOR	204	16	0	0
ISM	60	133	21	6
ISE	21	18	175	6
VPB	0	2	40	130

Results provided by proposed methods are comparable to that reported by other authors in the last decade (some examples are shown in Table 4). Particularly, linear DFA seems to be the least suitable for heartbeat classification according to previous studies as well as above results. Other models, such as NB, k-NN and SVM provide similar or even lower classification performance as compared to this study. SVM and k-NN provide the most successful results among other classifiers (such as in the present study) and are probably the most widely used in this area.

**Table 4:** Heartbeat classification methods. ISCH – ischemic heartbeats; PTCA - percutaneous transluminal coronary angioplasty; Se, Se, Acc – sensitivity, specificity and accuracy, respectively.

Type of heartbeats	Features	Classifier	Classification performance	Authors
NOR, particular minutes of ISCH	Morphological (QRS loop, ST-change vector magnitude, spatial ventricular gradient features)	Linear DFA	Se = 95.4%	Correa *et al*.^3^
			Sp = 95.2 (for the 4^th^ min of the PTCA)	
NOR, mild ISCH	High-frequency QRS	Logistic regression	Se = 46%, Sp = 87%	Sharir *et al*.^37^
NOR, moderate/severe ISCH			Se = 69%, Sp = 86%	
NOR, mild ISCH	Morphological (Q and R amplitudes, QRS slopes and energy, repolarization duration, normalized ST, etc.)	Logistic regression	Se = 77%, Sp = 88%	Llamedo *et al*. 2013 cinc^38^
NOR, moderate/severe ISCH			Se = 76%, Sp = 90%	
NOR, ISCH	Morphological (QRS slopes)	Linear DFA	Se = 24%, Sp = 93%	Firoozabadi *et al*.^4^
NOR, ISCH	Morphological (samples of heartbeats)	k-NN, SVM	Acc(k-NN) = 94.6%	Murthy *et al*.^5^
			Acc(linear SVM)=91.5%	
			Acc(RBF SVM)=95.3%	
NOR, ISCH	Morphological	SVM	Se = 94.8%, Sp = 99.5%	Tseng *et al*.^6^
NOR, VPB and 2 others	Morphological, RR intervals, higher-order statistics	Linear DFA,	DFA: Acc(NOR)=88.6%	Doquire *et al*.^10^
		SVM	Acc(VPB)=80.6%	
			SVM: Acc(NOR)=75.9%	
			Acc(VPB)=85.1%	
NOR, VPB and 1 other	AR model coefficients and non-linear features	Linear DFA	Acc up to 88%	Balli *et al*.^9^
NOR, VPB	Morphological	SVM	Acc = 88.9%	Alajlan *et al*.^7^
NOR,VPB + 3 others (except ISCH)	Higher order statistics of wavelet coefficients	k-NN	Se(NOR) = 97.5%	Kutlu *et al*.^12^
			Sp(NOR) = 95.3%	
			Se(VPB) = 87.6%	
			Sp(VPB) = 96.8%	
NOR, VPB + 5 others (except ISCH)	Spectral	k-NN	Acc = 97.0%	Arif *et al*.^8^
NOR,VPB + 3 others (except ISCH)	Higher order statistics	k-NN	Se(NOR) = 100%	Karimifard *et al*.^11^
			Sp(NOR) = 99.9%	
			Se(VPB) = 94.1%	
			Sp(VPB) = 99.8%	
NOR, VPB	Kalman filter, polarogram	NB	Acc = 98.8%	Sayadi *et al*.^13^
NOR, VPB+10 others (except ISCH)	Morphological, spectral and statistic	SVM	Acc = 98.9%	Shen *et al*.^14^
NOR, ISM, ISE, VPB	Morphological (*AreaD*)	k-NN	Acc = 98.6%	Proposed method
NOR, ISM, ISE, VPB	Morphological (*MorphD*)	SVM	Acc = 93.5%	Proposed method

However, it should be noted that recently used classification approaches differ from each other in various aspects. Particularly, number and types of analysed heartbeats vary among different studies (see Table 4). To the best of our knowledge, none of reported multi-class methods provides the discrimination among particular phases of ischemia. The only studies focused on the detection of particular time of ischemia (minute-by-minute approach^3^ or middle and severe phases of ischemia^37, 38^, utilize separate binary classifiers. Instead, common approaches (e.g. refs 4–6) are based on binary classification of data from healthy subjects and those with ischemia diagnosed by standard guidelines^23^. Binary models can also be used for distinguishing between NOR and VPBs^7, 13^ However, various types of arrhythmias including VPB are usually recognized in ECG by multi-class approaches^8–12, 14^.

Present study, on the contrary, introduces for the first time 4-class methods for classification of non-pathologic heartbeats, those with moderate and severe ischemia manifestations and concomitant VPBs. Besides abovementioned, many other factors, such as data used for analysis (input electrophysiological signals as well as computed features), settings and types of classification models, method for performance evaluation, etc. – vary among different approaches. Thus, direct comparison of reported results with each other and with our observations is rather difficult.

## Conclusion

Accurate detection of pathological events in ECG and their classification is important for correct diagnosis of cardiac disorders and further treatment of the patients. Therefore, this research area is highly topical for the past few decades. This paper introduces the results of comparative study, where the adequacy of various features as well as classification models for recognizing four different heartbeat types derived from experimental ECG was proven. The use of experimental records from animal isolated hearts enabled collecting unique dataset, which consists of heartbeats representing two different grades of myocardial ischemia (moderate and severe) and subsequent VPBs. Discrimination among pathological heartbeats is rather difficult because of their patterns’ similarity.

In the present work, this challenging task was solved by various classification approaches combining various morphological and spectral features with four classification methods. In contrast to other studies, examination of accuracy of the approaches was systematically performed on a single dataset, which allows direct comparison. Our results show that morphological features are more appropriate than spectral ones. Taking into account low computational complexity of morphological features calculation and simplicity of their interpretation by clinicians, above observation might be attractive for designing of automatic or semi-automatic ECG-based diagnostic systems. Furthermore, use of ECG features common in clinical practice or similar to them allows easy verification of computer-based diagnostic results by human expert and elimination of misinterpretations caused by automatic algorithm imperfection.

Among evaluated classification models, k-NN and RBF SVM seem to be the most suitable ones, even if reduced number of features is involved in training process. The proposed classification approaches allow achieving performance (Acc up to 98.6% with both Se and Sp higher than 95% for each heartbeat type) comparable with previously published similar methods or even surpassing them. Results also demonstrate that time-consuming delineation of QRS-T segment is not necessary for calculating the features capable of recognizing heartbeat types. This observation might be especially important in designing of systems for real-time ECG classification, where creation of simple, fast and accurate algorithms suitable for timely diagnosis is necessary.

Under conditions of continuous technological and economic progress and rapid development of new tools such as telemedicine, the amount of patients’ data needed to be analysed is growing exponentially. Despite the experimental character of this study, the main issues addressed can be considered relevant for clinical practice due to similarities in characteristics of rabbit and human hearts. Thus, the results contribute to multidisciplinary field focusing on creation of robust and reliable computer-based diagnostic systems, able to substitute the time-consuming human scoring.
